# Assessment of Brain Tumour Perfusion Using Early-Phase ^18^F-FET PET: Comparison with Perfusion-Weighted MRI

**DOI:** 10.1007/s11307-023-01861-2

**Published:** 2023-10-17

**Authors:** Christian P. Filss, Julian Cramer, Saskia Löher, Philipp Lohmann, Gabriele Stoffels, Carina Stegmayr, Martin Kocher, Alexander Heinzel, Norbert Galldiks, Hans J. Wittsack, Michael Sabel, Bernd Neumaier, Jürgen Scheins, N. Jon Shah, Philipp T. Meyer, Felix M. Mottaghy, Karl-Josef Langen

**Affiliations:** 1 Department of Nuclear Medicine, RWTH University Hospital, Aachen, Germany; 2 Institute of Neuroscience and Medicine (INM-3, INM-4, INM-5, INM-11), Forschungszentrum Jülich, Jülich, Germany; 3 Center of Integrated Oncology (CIO), University of Aachen, Bonn, Cologne and Düsseldorf, Germany; 4 Faculty of Medical Engineering and Technomathematics, FH Aachen University of Applied Sciences, Campus Juelich, Jülich, Germany; 5 Department of Stereotactic and Functional Neurosurgery, Center for Neurosurgery, University Hospital Cologne, Cologne, Germany; 6 Department of Nuclear Medicine, University Hospital Halle (Saale), Halle (Saale), Germany; 7 Department of Neurology, University Hospital Cologne, Cologne, Germany; 8 Department of Diagnostic and Interventional Radiology, Medical Faculty, University of Düsseldorf, Düsseldorf, Germany; 9 Department of Neurosurgery, University Hospital Düsseldorf, Düsseldorf, Germany; 10 Institute of Radiochemistry and Experimental Molecular Imaging, University Hospital Cologne, Cologne, Germany; 11 JARA - BRAIN - Translational Medicine, RWTH Aachen University, Aachen, Germany; 12 Department of Neurology, RWTH Aachen University Hospital, Aachen, Germany; 13 Department of Nuclear Medicine, Medical Center - University of Freiburg, Faculty of Medicine, University of Freiburg, Freiburg, Germany; 14 Department of Radiology and Nuclear Medicine, Maastricht University Medical Center (MUMC+), Maastricht, Netherlands

**Keywords:** Brain tumour, Glioma, PWI, rCBV, Early FET PET, Glioma, O-(2-18F-Fluoroethyl)-L-tyrosine, Amino acid PET

## Abstract

**Purpose:**

Morphological imaging using MRI is essential for brain tumour diagnostics. Dynamic susceptibility contrast (DSC) perfusion-weighted MRI (PWI), as well as amino acid PET, may provide additional information in ambiguous cases. Since PWI is often unavailable in patients referred for amino acid PET, we explored whether maps of relative cerebral blood volume (rCBV) in brain tumours can be extracted from the early phase of PET using *O*-(2-^18^F-fluoroethyl)-L-tyrosine (^18^F-FET).

**Procedure:**

Using a hybrid brain PET/MRI scanner, PWI and dynamic ^18^F-FET PET were performed in 33 patients with cerebral glioma and four patients with highly vascularized meningioma. The time interval from 0 to 2 min p.i. was selected to best reflect the blood pool phase in ^18^F-FET PET. For each patient, maps of MR-rCBV, early ^18^F-FET PET (0–2 min p.i.) and late ^18^F-FET PET (20–40 min p.i.) were generated and coregistered. Volumes of interest were placed on the tumour (VOI-TU) and normal-appearing brain (VOI-REF). The correlation between tumour-to-brain ratios (TBR) of the different parameters was analysed. In addition, three independent observers evaluated MR-rCBV and early ^18^F-FET maps (^18^F-FET-rCBV) for concordance in signal intensity, tumour extent and intratumoural distribution.

**Results:**

TBRs calculated from MR-rCBV and ^18^F-FET-rCBV showed a significant correlation (*r* = 0.89, *p* < 0.001), while there was no correlation between late ^18^F-FET PET and MR-rCBV (*r* = 0.24, *p* = 0.16) and ^18^F-FET-rCBV (*r* = 0.27, *p* = 0.11). Visual rating yielded widely agreeing findings or only minor differences between MR-rCBV maps and ^18^F-FET-rCBV maps in 93 % of the tumours (range of three independent raters 91–94%, kappa among raters 0.78–1.0).

**Conclusion:**

Early ^18^F-FET maps (0–2 min p.i.) in gliomas provide similar information to MR-rCBV maps and may be helpful when PWI is not possible or available. Further studies in gliomas are needed to evaluate whether ^18^F-FET-rCBV provides the same clinical information as MR-rCBV.

**Supplementary Information:**

The online version contains supplementary material available at 10.1007/s11307-023-01861-2.

## Background

Standard imaging of brain tumours includes anatomical MRI with T1-weighted images pre (T1) and post-contrast enhancement (T1c) and T2-weighted/FLAIR images [1]. Differentiating tumour progression (TP) from treatment-related changes (TRC) after surgery, radiotherapy and chemotherapy, however, may be challenging because contrast enhancement in MRI is not specific to neoplastic tissue [1, 2].

In order to improve diagnostic accuracy in pretreated and recurrent brain tumours, dynamic susceptibility contrast (DSC) perfusion-weighted MRI (PWI) is frequently performed using the relative cerebral blood volume (rCBV) as the most sensitive parameter for vascularity [2]. Another important approach to differentiate TP and TRC is PET using radiolabelled amino acids, as recommended by the PET Response Assessment in Neuro-Oncology (RANO working group) [3]. Both methods provide information on tumour biology that is complementary to morphological MRI and are especially helpful in the differentiation of TP and TRC in pretreated gliomas [4–6].

Since rCBV and amino acid uptake represent different physiological parameters, the extent and regional distribution of the signal changes differ substantially [4, 5, 7–10]. Whether the combination of rCBV mapping and amino acid PET imaging increases accuracy in differentiating TP or TRC or whether a sequential use of both techniques is more reasonable remains a controversial question [11–14].

In our department, PET data using the amino acid PET tracer O-(2-^18^F-fluoroethyl)-L-tyrosine (^18^F-FET) are available for several thousand patients, but data on rCBV mapping using PWI are frequently not available [15]. Of note, PET has long been used to measure rCBV in both normal and abnormal tissue, including cancer [16]. The optimal way to measure rCBV using PET is with ^15^O-labelled carbon monoxide [17]. In principle, however, rCBV can be measured with any PET radiotracer administered intravenously as long as data acquisition begins at the time of injection and the tracer is slowly extracted from the blood pool [16]. Dynamic acquisition is part of the standard protocol used at the Forschungszentrum Jülich, and time-activity curves in the tumour provide additional information for grading and for differential diagnosis of brain lesions [18–23]. We hypothesized that the first minutes of the dynamic PET scans available from the existing database contain the data necessary to evaluate rCBV.

The aim of this study was to explore whether imaging of early-phase ^18^F-FET uptake after injection in patients with brain tumours provides information similar to that of MR-rCBV provided by DSC PWI. This could provide an option to evaluate the additive value of ^18^F-FET-rCBV when combined with late ^18^F-FET uptake in different diagnostic questions. For this purpose, the data of patients who underwent simultaneous PWI and dynamic ^18^F-FET PET in a previous hybrid PET MRI study were analysed retrospectively [5]. Other studies have used the term “early ^18^F-FET PET” to describe the early phase of amino acid uptake from 5–15 min after injection [24–26]. In order to avoid confusion, we refer to ^18^F-FET imaging in the immediate phase after injection (0–2 min) in the following as ^18^F-FET-rCBV.

## Methods

### Patient Population

Thirty-three patients with histologically characterized glioma, according to the classification of the World Health Organization (WHO) of Tumours of the Central Nervous System of 2007 [27], investigated using a hybrid PET/MR scanner between February 2011 and January 2013, were included in this study. A newer tumour classification is not available for this patient collective but is not relevant to the question investigated. Three patients had a WHO grade II astrocytoma, four patients had a WHO grade III anaplastic astrocytoma, two patients had a WHO grade II oligoastrocytoma, three patients had a WHO grade III anaplastic oligoastrocytoma, four patients had a WHO grade oligodendroglioma, one patient had a WHO grade III ependymoma, and 16 patients had a WHO grade IV glioblastoma (*n*=21 untreated, *n*=12 pretreated, 17 women and 16 men, mean age 48, age range 25–75 years) [27]. In addition, data from four patients with highly vascularized meningiomas in MR-rCBV were included to determine the optimal time window for rCBV assessment in ^18^F-FET PET. The clinical data of the patients and the results of the different imaging parameters are shown in Supplemental Tables 1 and 2. The patient data were part of a previously published study investigating the relationship between MR-rCBV and late ^18^F-FET uptake [5]. The Ethics Committee of the University of Düsseldorf approved the hybrid PET-MRI investigations (study numbers 3167 and 2438). All subjects gave written informed consent for their participation prior to the study.

### MR Imaging

MRI was performed using a Siemens 3T Magnetom Trio MR scanner. Anatomical MRI included a T1-weighted MPRAGE sequence (T1), T2-weighted FLAIR sequence (FLAIR) and contrast-enhanced T1-weighted MPRAGE sequence (T1c) conducted 3 min after injection of the contrast agent gadoteric acid (DOTAREM; Guerbet) with a dose of 0.1–0.2 mmol/kg body weight. A dynamic susceptibility-weighted contrast-enhanced T2* sequence (DSC) measuring the first pass of a contrast agent bolus (single shot echo planar imaging sequence (EPI) was used for PWI: dynamic interscan interval = 1500 ms; echo time (TE) =32 ms; flip angle = 90°, image matrix = 128 × 128, field of view FOV = 230 mm × 230 mm, slice thickness 5 mm). The contrast agent was injected with a power injector Injektron 82 MRT (Medtron AG) via an 18–20-gauge intravenous catheter at a dose of 0.1 mmol/kg body weight (flow rate, 5 ml/s). Parametric rCBV maps were created from DSC MRI data using the software Stroketool version 2.7 [28].

### PET Imaging

The amino acid ^18^F-FET was produced and applied as described previously [29]. Dynamic PET scans were acquired for 40 min after the manual intravenous injection of a bolus of approximately 3 MBq ^18^F-FET/ kg body weight followed by flush of 10 ml saline solution. PET imaging was performed simultaneously with MR imaging using a brain PET insert. The brain PET is a compact cylinder that fits in the bore of the Siemens 3T Magnetom Trio MR scanner (axial FOV of 19.2 cm, optimum spatial resolution of 3-mm full-width at half maximum) [30]. The list mode PET data were reconstructed into 14 time frames (5 × 1 min, 5 × 3 min and 4 × 5 min) using OP-OSEM. Data were corrected for random, scattered coincidences, deadtime and attenuation. Attenuation correction was based on a template-based approach [31]. The reconstructed dynamic dataset was smoothened using a 3-mm 3D Gaussian filter kernel. ^18^F-FET uptake in the tissue was expressed as a standardized uptake value (SUV) by dividing the radioactivity concentration (kBq/ml) in the tissue by the radioactivity injected per gramme of body weight. ^18^F-FET PET images from 20 to 40 min p.i. were summed up for standard late imaging.

To identify the optimal time window of the blood pool phase in ^18^F-FET PET, an averaged time-activity curve (TAC) of ^18^F-FET uptake in four highly vascularized meningiomas was generated (Supplemental Figure 1). The time window of 0–2 min after injection captured the early peak after tracer injection and was defined as best reflecting the blood pool phase in ^18^F-FET PET. Consequently, this time window was used for the generation of ^18^F-FET-rCBV maps (0–2 min p.i.).

### Data Analysis

Prior to further processing, the anatomical MRI, MR-rCBV maps, ^18^F-FET PET rCBV maps and late ^18^F-FET PET images were coregistered using the software PMOD (version 4.102; PMOD Ltd.). Together with anatomical MRI (T1, T1c, FLAIR), late ^18^F-FET PET images were used to identify the gross tumour region.

Spherical VOIs with a diameter of 16 mm were placed in the centre of the most pronounced signal changes in the gross tumour region in the MR-rCBV maps, as described previously [32]. In cases where the MR-rCBV maps did not show relevant signal alterations in the gross tumour area, anatomical MRI and late ^18^F-FET PET were used to define the centre of the tumour VOI. Particular care was taken to avoid large vessels in the VOIs. From these tumour VOIs, the mean MR-rCBV, mean ^18^F-FET PET rCBV and mean late ^18^F-FET uptake were determined. A larger reference VOI with a diameter of 30 mm was placed in the normal-appearing brain tissue in the hemisphere contralateral to the gross tumour region at the level of the ventricles, including both white and grey matter. The location of the VOI was checked in all other images to ensure a representative background and to avoid artefacts (e.g. large vessels). Mean tumour-to-brain ratios (TBRs) were calculated by dividing the mean value of the respective parameter in the tumour VOI by the corresponding mean value of the reference VOI [33–35]. Additionally, a histogram analysis was performed in 4 representative glioma patients (Pat. 10, 20, 21 and 23). In these patients, additional banana shaped ROI’s were placed in the grey matter and in the white matter at the level of the centrum semiovale.

### Visual Comparison of MR-rCBV and^18^F-FET-rCBV Maps

Maps of MR-rCBV-maps and ^18^F-FET-rCBV were compared visually in terms of signal intensity, extent and regional variability. The comparison was made by three independent investigators experienced in reading MR-rCBV and ^18^F-FET PET scans (K-JL, PL and CF). Each investigator assigned the signal abnormalities in the tumour area in the different maps to one of the following categories: (1) widely agreeing, (2) minor differences, (3) major differences and (4) disagreeing results. Furthermore, MR-rCBV-maps and ^18^F-FET-rCBV maps were compared with standard late ^18^F-FET images (20–40’).

### Statistics

The Pearson correlation analysis was used for correction analysis. Probability values less than 0.05 were considered statistically significant. Bland–Altman analysis was performed to compare rCBV measured with MR-rCBV and ^18^F-FET-rCBV. The Cohen’s κ coefficient was used to measure the degree of inter-rater agreement for visual comparison and the assignment of MR-rCBV and ^18^F-FET-rCBV maps to different categories of similarity: κ values between 0 and 0.20 were considered to indicate a positive but slight agreement, between 0.21 and 0.40 a fair agreement, between 0.41 and 0.60 a good agreement, between 0.61 and 0.80 a very good agreement and greater than 0.80 an excellent agreement.

## Results

### Pearson Correlation Analysis

**Fig. 1:**
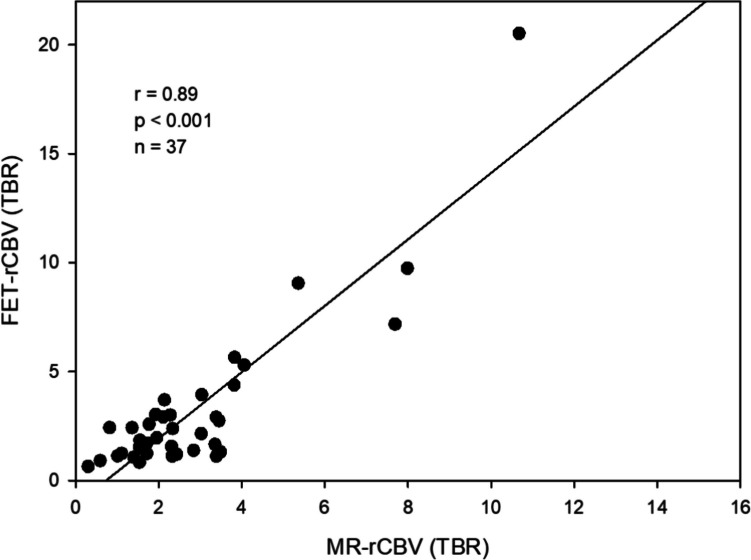
Statistically significant correlation between MR-rCBV (TBR) and ^18^F-FET PET-rCBV (TBR) in 37 brain tumours (33 patients with cerebral gliomas and four patients with meningiomas) indicating comparable findings

**Fig. 2:**
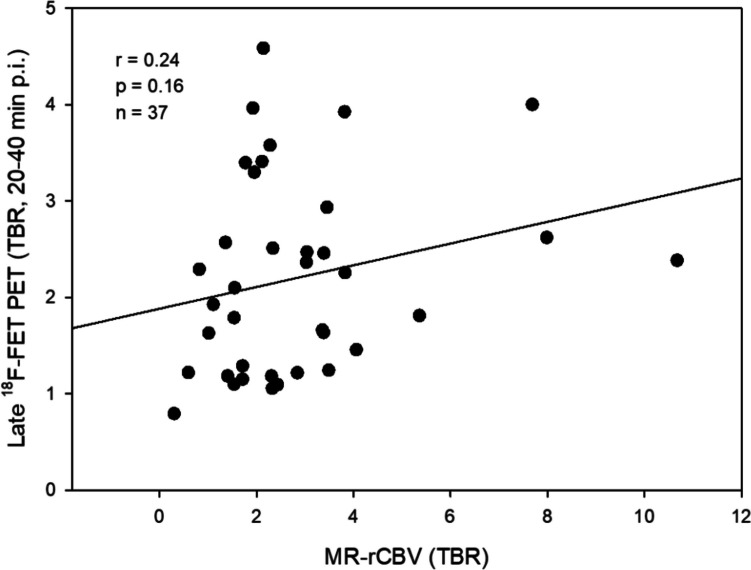
Relationship of MR-rCBV (TBR) and late ^18^F-FET uptake (TBR, 20–40 min p.i.) in 37 brain tumours (33 patients with cerebral gliomas and four patients with meningiomas). No correlation was observed between the two parameters

The TBRs of MR-rCBV and ^18^F-FET-rCBV of gliomas showed a significant correlation (*r* = 0.89, *p* < 0.001) (Figure 1). In contrast, there was no correlation between TBRs of MR-rCBV and late ^18^F-FET uptake (*r* = 0.24, *p* = 0.16) (Figure 2) and no correlation between TBRs of ^18^F-FET-rCBV and standard late ^18^F-FET uptake (*r* = 0.27, *p* = 0.11).

### Bland–Altman Analysis

Bland–Altman analysis revealed that measuring rCBV with MR-rCBV and ^18^F-FET-rCBV showed a general good agreement between the two methods with the mean difference = −0.4 indicating a small bias to higher values in ^18^F-FET-rCBV compared to MR-rCBV and especially one outlier with very high values in both modalities (Supplemental Figure 2).

### Visual Evaluation and Inter-rater Agreement

Visual rating yielded broadly consistent or minor differences (category 1 and 2) in the tumour regions between the MR-rCBV and ^18^F-FET-rCBV maps in 93 % of the cases (range 91–94%). The evaluation of the inter-rater agreement showed an excellent agreement between the raters, with a mean κ value of 0.85 (range, 0.78–1.0).

**Fig. 3:**
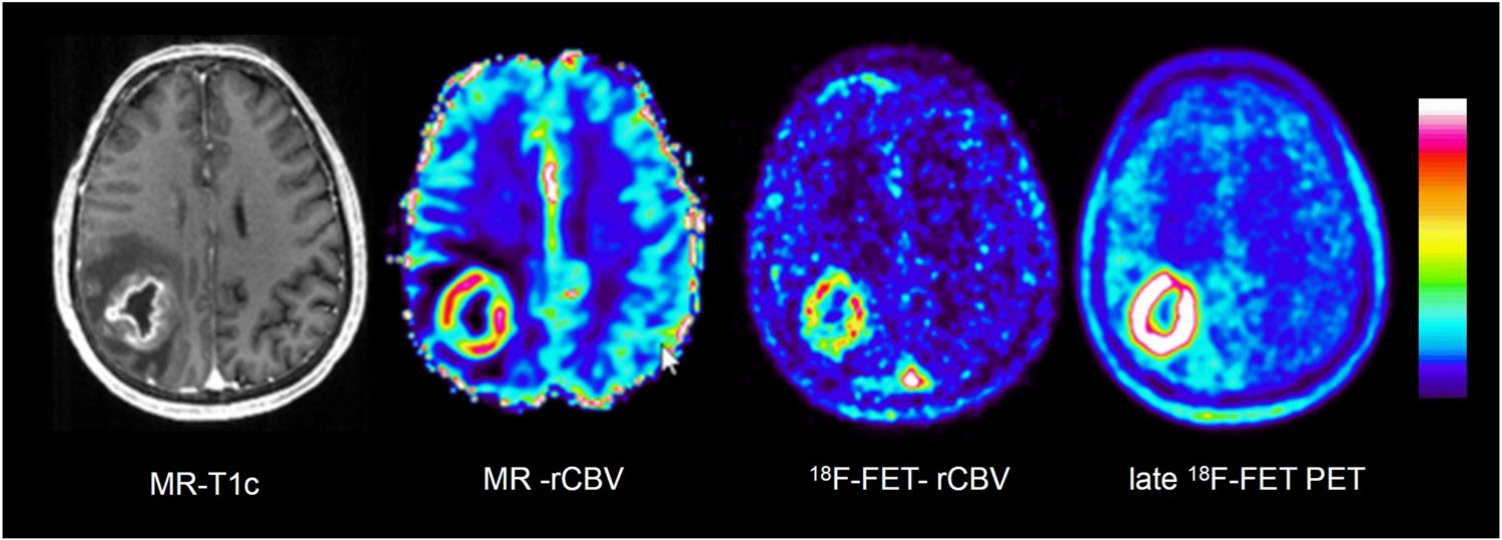
Contrast-enhanced T1-weighted MRI (MR-T1c), MR-rCBV, ^18^F-FET-rCBV and late ^18^F-FET PET (from left to right) in a patient with a newly diagnosed glioblastoma. There is a ring-enhancing lesion in the right parietal cortex showing a pronounced signal in MR-rCBV, ^18^F-FET-rCBV and late ^18^F-FET PET. The findings of MR-rCBV and ^18^F-FET-rCBV are very similar

**Fig. 4:**
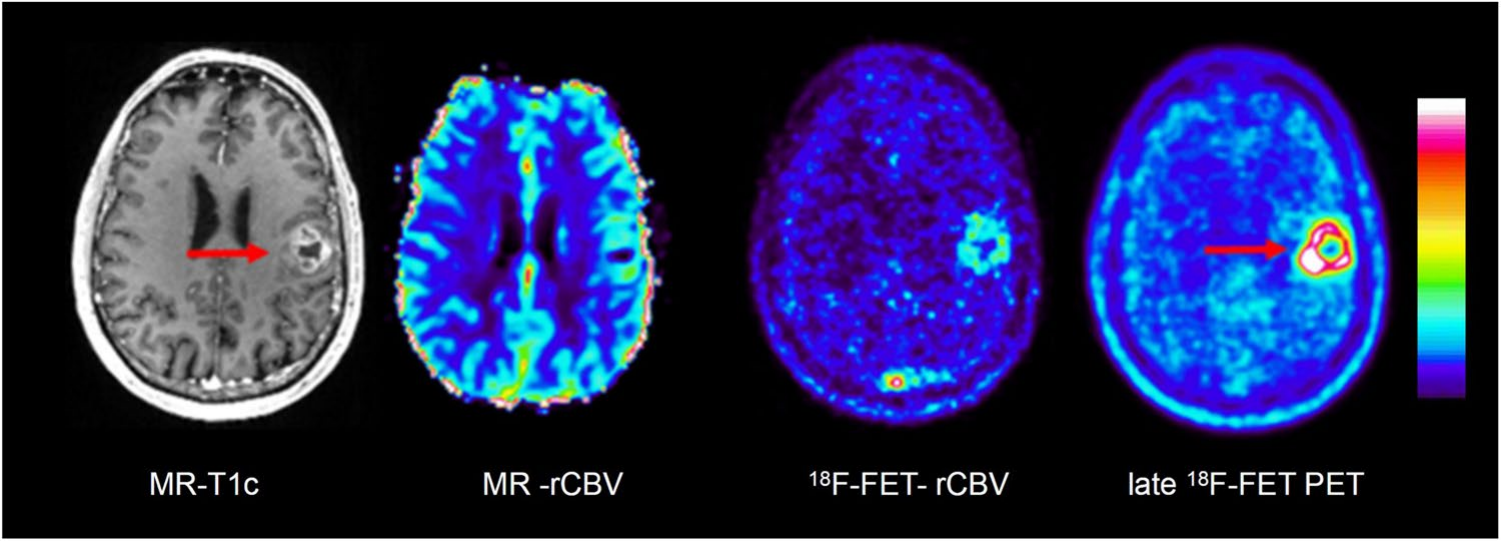
Contrast-enhanced T1-weighted MRI (MR-T1c), MR-rCBV, ^18^F-FET-rCBV and late ^18^F-FET PET (from left to right) in a patient with a newly diagnosed glioblastoma. There is a contrast-enhancing lesion in the left frontoparietal cortex showing a pronounced signal on late ^18^F-FET PET (red arrows) but only a discrete signal in MR-rCBV and ^18^F-FET-rCBV. The findings of MR-rCBV and ^18^F-FET-rCBV are very similar

**Fig. 5:**
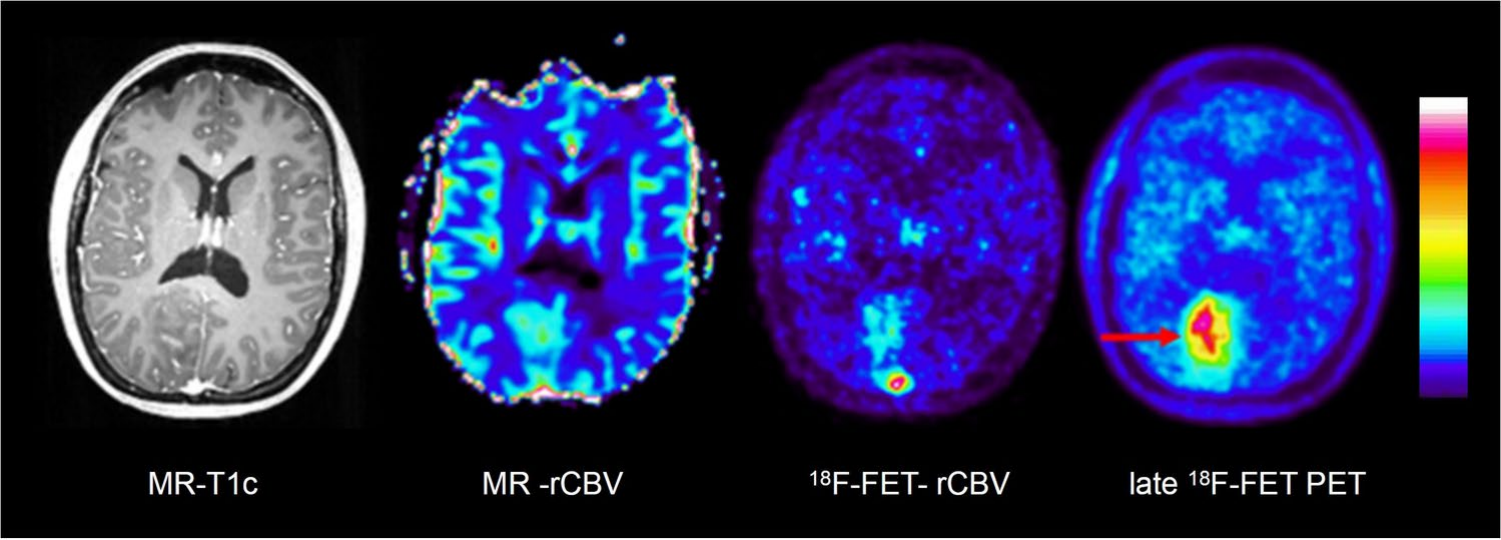
Contrast-enhanced T1-weighted MRI (MR-T1c), MR-rCBV, ^18^F-FET-rCBV and late ^18^F-FET PET (from left to right) in a patient with an untreated oligodendroglioma WHO grade II. There is no relevant contrast enhancement in MRI but pronounced tracer uptake in the right parietal cortex in late ^18^F-FET PET (red arrow). In contrast, there is only a weak signal in MR-rCBV and ^18^F-FET-rCBV. The findings of MR-rCBV and ^18^F-FET-rCBV are very similar

**Fig. 6:**
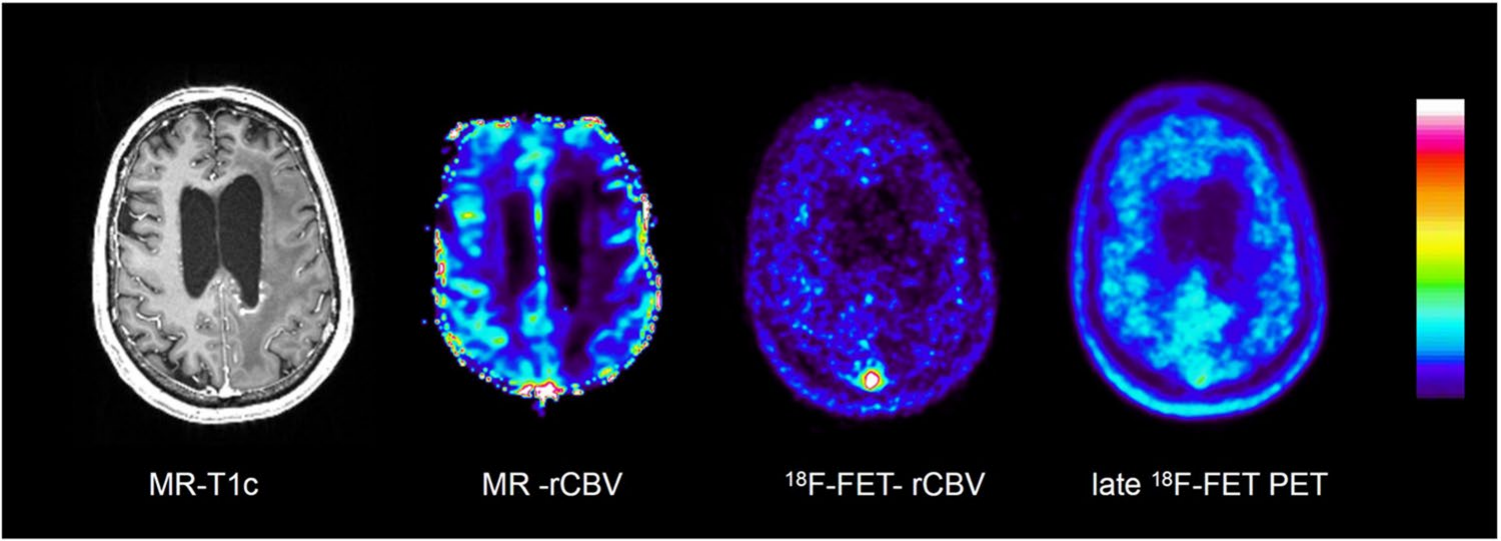
Contrast-enhanced T1-weighted MRI (MR-T1c), MR-rCBV, ^18^F-FET-rCBV and late ^18^F-FET PET (from left to right) in a patient with an anaplastic astrocytoma (WHO grade III) after radio- and chemotherapy showing left temporo-occipital contrast enhancement on MR-T1c but no signal on MR-rCBV, ^18^F-FET-rCBV and late ^18^F-FET PET. The findings of MR-rCBV and ^18^F-FET-rCBV are very similar

Figures 3, 4, 5 and 6 show representative examples of contrast-enhanced T1-weighted MRI, MR-rCBV, ^18^F-FET-rCBV maps and standard late ^18^F-FET PET images in patients with cerebral gliomas. Figure 3 demonstrates the case of a glioblastoma patient with a pronounced signal in MR-rCBV, ^18^F-FET-rCBV and late ^18^F-FET PET. Figures 4 and 5 show a glioblastoma and an oligodendroglioma WHO grade II with pronounced ^18^F-FET PET uptake but only a moderately increased signal in MR-rCBV and ^18^F-FET-rCBV. Figure 6 shows images of a patient with an anaplastic astrocytoma WHO grade III with contrast enhancement in T1-weighted MRI but no signal in MR-rCBV and ^18^F-FET-rCBV and late ^18^F-FET PET. In all cases, the findings of MR-rCBV and ^18^F-FET-rCBV are highly comparable.

### Histogram Analysis

The histograms for MR-rCBV and ^18^F-FET-rCBV in the tumour and grey and white matter of 4 representative glioma patients are shown in Supplemental Figure 3. Both MR-rCBV and ^18^F-FET-rCBV showed a good separation of tumour and grey and white matter.

## Discussion

This study demonstrates that imaging of ^18^F-FET uptake within the first 2 min after injection in patients with brain tumours provides information similar to that obtained with MR-rCBV. Although rCBV maps provided by DSC PWI cannot be considered a gold standard in the same way as PET using ^15^O-labelled carbon monoxide [17], it constitutes a robust marker for rCBV that is widely used and accepted in clinical practice [1]. However, PWI is often not available in patients referred for ^18^F-FET PET or, depending on the location of the tumour, is prone to susceptibility artefacts. Here, rCBV extracted from ^18^F-FET PET might be a useful alternative. Since a number of centres have collected dynamic data sets with amino acid PET in several thousand patients over the past 20 years, this would allow to retrospectively investigate the benefit of combining rCBV and amino acid uptake in different clinical settings. This includes molecular characterization of untreated brain tumours, differentiation of tumour progression and therapy-related changes and therapy monitoring. This approach is not limited to ^18^F-FET PET but should be equally applicable to other amino acid tracers such as 3,4-dihydroxy-6-[^18^F]-fluoro-L-phenylalanine (FDOPA) or anti-1-amino-3-[^18^F]fluorocyclobutane-1-carboxylic acid (FACBC or fluciclovine) [36].

Regarding the use of ^18^F-FET PET to estimate rCBV, some basic aspects have to be discussed.

In our standard ^18^F-FET PET protocol, the tracer is injected manually as an intravenous bolus, which is more susceptible to delay and dispersion compared to the bolus generated by the power injector required for DSC. In order to determine the optimal time interval to image ^18^F-FET-rCBV, we analysed the time-activity curves of four highly vascularized meningiomas, which exhibited a strong signal in MR-rCBV maps in a previous study [5]. The mean time-activity curves of the meningiomas showed an early peak in the time interval 0–2 min post-injection, which appeared to be well suited for rCBV evaluation (Supplemental Figure 1). ^18^F-FET is an amino acid that is transported into the brain and into brain tumour tissue by facilitated transport via large neutral amino acid transporters (subtypes LAT1 and LAT2) [37]. First-pass extraction of ^18^F-FET is low, and the peak of the time-activity curve in malignant gliomas is usually later than 5 min after injection and later than 40 min in lower-grade gliomas [19]. Therefore, it is very unlikely that the tracer signal during the first 2 min after injection originates from a compartment other than the vascular pool. In line with this assumption, there is a clear visualization of the large vessels, such as the sagittal sinus, in the ^18^F-FET-rCBV maps from this time window (Figures 3, 4, 5 and 6). Although disruption of the blood–brain barrier in the tumour area may cause an non-specific signal, the fact that there is a low ^18^F-FET-rCBV signal in several contrast-enhancing tumours in this series of patients indicates that ^18^F-FET-rCBV is unlikely to influenced by blood–brain barrier integrity.

Furthermore, no correlation between the TBRs of ^18^F-FET-rCBV and the TBRs of late ^18^F-FET uptake (Figure 2) was observed, which indicates a difference between the two measurements and is in line with previous publications demonstrating that late amino acid uptake is more strongly correlated with cell density than with tumour vascularity [5, 38, 39].

Visual rating by different raters yielded widely agreeing findings or minor differences between MR-rCBV and ^18^F-FET-rCBV maps in 93 % of the cases. Small differences between the images are to be expected, especially since MR-rCBV is a parametric image calculated from the time-activity curve in individual pixels and may be more susceptible to artefacts than a tracer distribution image. The histograms for MR-rCBV and ^18^F-FET-rCBV in the tumour and grey and white matter of 4 representative glioma patients were comparable and showed a good separation of tumour and grey and white matter (Supplemental Figure 3), which is in line with the visual impression.

Finally, some limitations of this study have to be considered. Firstly, the number of patients is too small to enable final conclusions to be drawn. Furthermore, the comparability of the MR-rCBV and FET-rCBV maps is limited by principal differences in the underlying imaging technologies. This leads to different kinds of artefacts, which have to be considered by the raters in their interpretation of the images. Consequently, the complementary value of rCBV and late ^18^F-FET in brain tumour diagnosis needs to be investigated in larger collectives of patients. A recent study reported promising results in this regard, demonstrating increased diagnostic accuracy by combining ^18^F-FET PET and perfusion- and diffusion-weighted MRI in patients with suspected glioma recurrence [13]. Furthermore, the patient collective is too small to analyse an influence additional factor such as contrast enhancement in MRI or gender as demonstrated for late ^18^F-FET uptake [40]. These aspects should be further investigated in future studies.

## Conclusion

The present study suggests that ^18^F-FET PET imaging in the first 2 min after tracer injection yields rCBV maps comparable to those obtained by PWI. Thus, ^18^F-FET-rCBV data may be used instead of MR-rCBV when PWI is not possible, not available or if the tumour is located in brain regions that are prone to susceptibility artefacts that would ordinarily hamper the generation and interpretation of MR-rCBV maps. The described method makes it possible to retrospectively investigate the clinical significance of the combination of rCBV and late amino acid uptake from existing large data sets.

### Supplementary Information

ESM 1 (PNG 21 kb)

ESM 2 (PNG 76 kb)

ESM 3 (TIFF 79.7 kb)

ESM 4 (DOCX 43.6 kb)

ESM 5 (DOCX 36.0 kb)

## Data Availability

The datasets used and/or analysed during the current study are available from the corresponding author on reasonable request.
