# Supplementary figures and images for: Assessment of Brain Tumour Perfusion Using Early-Phase 18F-FET PET: Comparison with Perfusion-Weighted MRI
Source: Mol Imaging Biol. 2023 Oct 17;26(1):36–44. doi: 10.1007/s11307-023-01861-2 (PMC10827807; doi:10.1007/s11307-023-01861-2)

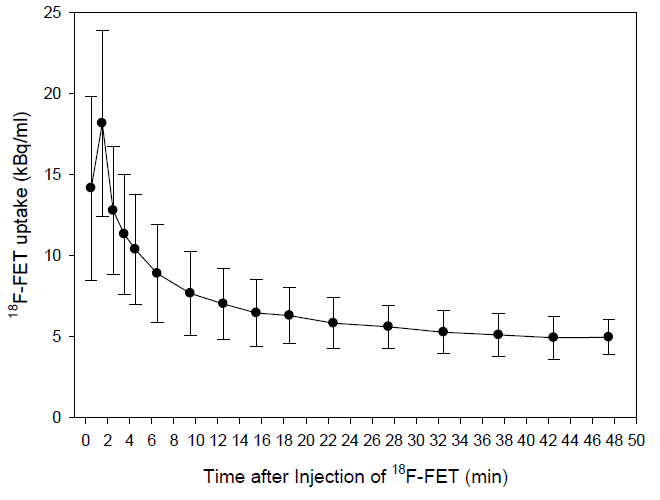

Supplement: Supplementary file 1 — (PNG 21 kb) [file 11307_2023_1861_MOESM1_ESM.png]

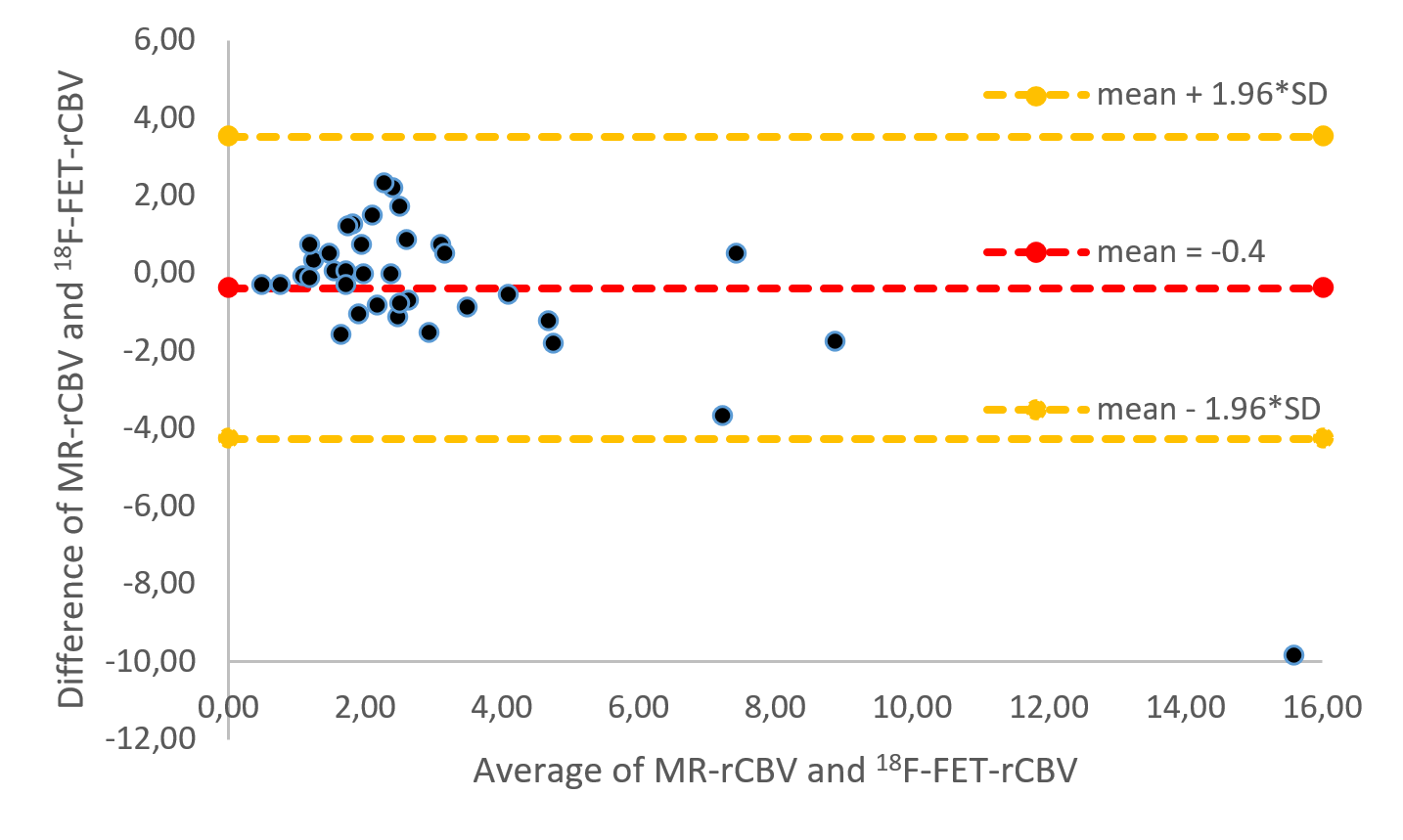

Supplement: Supplementary file 2 — (PNG 76 kb) [file 11307_2023_1861_MOESM2_ESM.png]

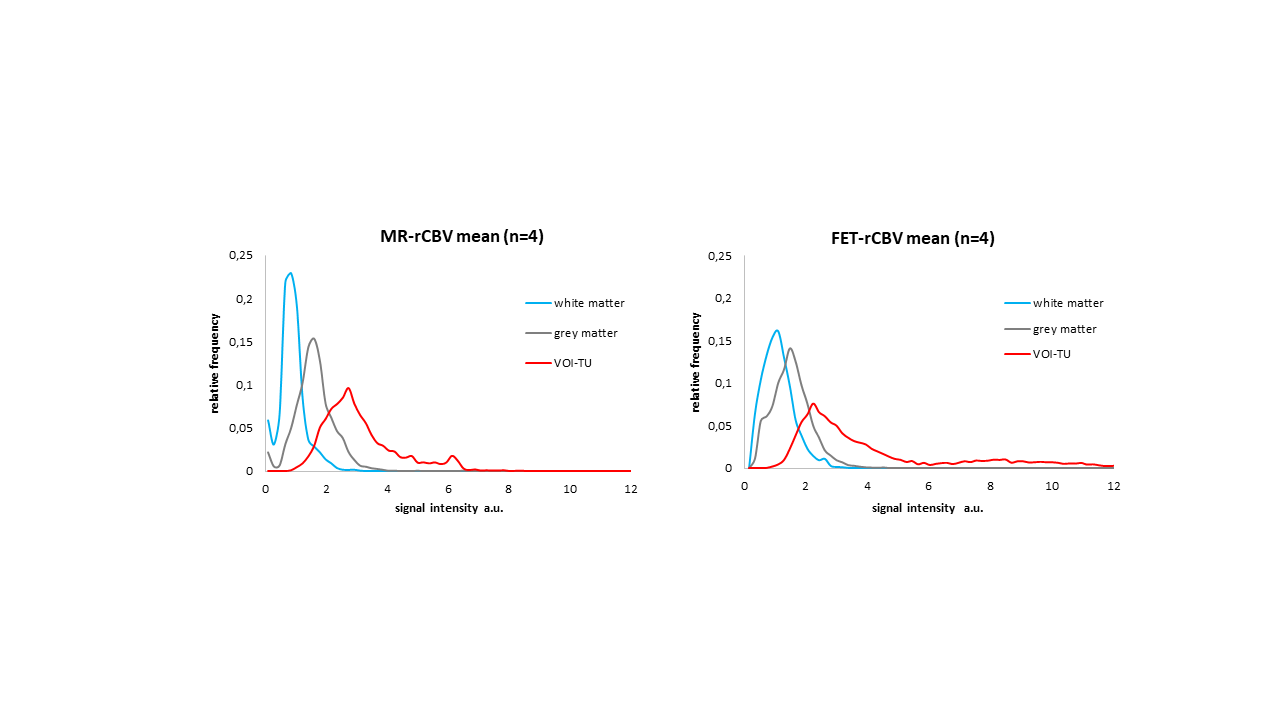

Supplement: Supplementary file 3 — (TIFF 79.7 kb) [file 11307_2023_1861_MOESM3_ESM.tif]
