# Supplementary material for: Assessment of Brain Tumour Perfusion Using Early-Phase 18F-FET PET: Comparison with Perfusion-Weighted MRI
Source: Mol Imaging Biol. 2023 Oct 17;26(1):36–44. doi: 10.1007/s11307-023-01861-2 (PMC10827807; doi:10.1007/s11307-023-01861-2)
Supplement: Supplementary file 4 — (DOCX 43.6 kb) [file 11307_2023_1861_MOESM4_ESM.docx]

**Supplemental Table 1: Clinical data and imaging parameters of glioma patients**

| **Pat. No.** | **Sex** | **Age** | **Location** | **Histology*** | **Pretreatment** | **CE in MRI** | **TBR_mean_** | | | **Visual Scoring** |
| --- | --- | --- | --- | --- | --- | --- | --- | --- | --- | --- |
|  |  |  |  |  |  |  | **MR- rCBV** | **FET-PET-rCBV** | **FET PET (20-40’)** |  |
| 1 | F | 49 | L P | A II | No | No | 1.40 | 1.07 | 1.18 | widely agreeing |
| 2 | M | 45 | L F | OA II | R, RTx | No | 1.95 | 1.97 | 3.30 | widely agreeing |
| 3 | M | 25 | L F-P | OA II | No | No | 0.30 | 0.65 | 0.79 | widely agreeing |
| 4 | F | 31 | R P | O II | No | Yes | 2.28 | 3.01 | 3.58 | widely agreeing |
| 5 | F | 59 | L T | A II | No | Yes | 1.71 | 1.24 | 1.15 | widely agreeing |
| 6 | F | 59 | L F | O II | No | Yes | 1.92 | 3.04 | 3.96 | widely agreeing |
| 7 | F | 51 | L T-O | A III | RTx, CTx | No | 1.01 | 1.13 | 1.63 | widely agreeing |
| 8 | M | 54 | L F | A II | No | Yes | 0.59 | 0.91 | 1.22 | widely agreeing |
| 9 | F | 49 | R T | O II | No | No | 0.82 | 2.43 | 2.29 | widely agreeing |
| 10 | F | 37 | R T-F | O II | No | Yes | 4.06 | 5.30 | 1.46 | widely agreeing |
| 11 | F | 69 | L I | E III | R | Yes | 1.54 | 1.54 | 1.79 | widely agreeing |
| 12 | F | 46 | L T | GBM | R, RTx, CTx | No | 2.31 | 1.57 | 1.18 | widely agreeing |
| 13 | M | 26 | L F | GBM | R, RTx, CTx | No | 2.43 | 1.21 | 1.09 | widely agreeing |
| 14 | F | 37 | R P | OA III | No | No | 2.84 | 1.39 | 1.22 | different |
| 15 | F | 53 | L T | GBM | R, RTx, CTx | Yes | 3.35 | 1.66 | 1.66 | widely agreeing |
| 16 | M | 52 | L P.O | GBM | R, RTx, CTx | Yes | 3.45 | 2.75 | 2.94 | widely agreeing |
| 17 | M | 75 | R T-P | GBM | No | Yes | 3.38 | 2.91 | 1.64 | widely agreeing |
| 18 | M | 59 | L I | GBM | No | Yes | 2.11 | 2.91 | 3.41 | widely agreeing |
| 19 | M | 41 | L T | OA III | R, RTx | Yes | 2.32 | 1.12 | 1.06 | widely agreeing |
| 20 | M | 58 | L F-P | GBM | No | Yes | 2.34 | 2.39 | 2.51 | widely agreeing |
| 21 | F | 42 | L T | GBM | No | Yes | 3.49 | 1.31 | 1.24 | different |
| 22 | M | 34 | R Li, L Li | A III | R, RTx, CTx | Yes | 3.02 | 2.16 | 2.36 | widely agreeing |
| 23 | F | 44 | R T, L I | GBM | R,RTx,CTx | Yes | 3.39 | 1.12 | 2.46 | widely agreeing |
| 24 | F | 32 | R P | GBM | No | Yes | 3.81 | 4.38 | 3.93 | widely agreeing |
| 25 | M | 66 | L I | GBM | No | Yes | 3.04 | 3.94 | 2.47 | widely agreeing |

**Supplemental Table 1: (continued)**

| **Pat. No.** | **Sex** | **Age** | **Location** | **Histology*** | **Pretreatment** | **CE in MRI** | **TBR_mean_** | | | **Visual Scoring** |
| --- | --- | --- | --- | --- | --- | --- | --- | --- | --- | --- |
|  |  |  |  |  |  |  | **MR- rCBV** | **FET-PET-rCBV** | **FET PET (20-40’)** |  |
| 26 | M | 50 | L P | GBM | No | Yes | 1.36 | 2.42 | 2.57 | widely agreeing |
| 27 | F | 73 | R P | GBM | No | Yes | 2.14 | 3.71 | 4.59 | widely agreeing |
| 28 | M | 61 | R T | A III | No | Yes | 1.71 | 1.72 | 1.29 | widely agreeing |
| 29 | M | 34 | R P | GBM | No | Yes | 1.53 | 0.84 | 1.10 | widely agreeing |
| 30 | F | 37 | R F | GBM | R,RTx,CTx | Yes | 1.55 | 1.85 | 2.10 | widely agreeing |
| 31 | M | 64 | R F | GBM | R, RTx, CTx | Yes | 1.11 | 1.25 | 1.93 | widely agreeing |
| 32 | F | 34 | R In | A III | No | Yes | 7.69 | 7.18 | 4.00 | widely agreeing |
| 33 | M | 46 | R F | OA III | No | No | 1.77 | 2.59 | 3.40 | widely agreeing |

**Legends Supplemental Table 1**

*sex: F = female, M = male*

*age in years at the time of the PET and MRI study*

*L = left hemispheric, R = right hemispheric, F = frontal, P= parietal, O = occipital, T = temporal, I = insular, Li = limbic*

*^*^ histological diagnosis: A II = astrocytoma WHO grade II, A III = anaplastic astrocytoma WHO grade III, O II = oligodendroglioma WHO grade II, OA II oligoastrocytoma WHO grade II, OA III = anaplastic oligoastrocytoma WHO grade III, E III = anaplastic ependymoma WHO grade III, GBM = glioblastoma WHO grade IV*

*Pretreatment before study: R = resection, RTx = radiation therapy, CTx = chemotherapy*

*CE in MRI = contrast enhancement in MRI*

*TBR_mean_ = mean tumour to brain ratio*

*Visual scoring: Mean value from 3 independent raters. Widely agreeing (Score 1-2), different (Score 3-4)*
