# Supplementary material for: Assessment of Brain Tumour Perfusion Using Early-Phase 18F-FET PET: Comparison with Perfusion-Weighted MRI
Source: Mol Imaging Biol. 2023 Oct 17;26(1):36–44. doi: 10.1007/s11307-023-01861-2 (PMC10827807; doi:10.1007/s11307-023-01861-2)
Supplement: Supplementary file 5 — (DOCX 36.0 kb) [file 11307_2023_1861_MOESM5_ESM.docx]

**Supplemental Table 2: Clinical data and imaging parameters of meningioma patients**

| **Pat. No.** | **Sex** | **Age** | **Location** | **Histology*** | **Pretreatment** | **CE in MRI** | **TBR_mean_** | | | **Visual Scoring** |
| --- | --- | --- | --- | --- | --- | --- | --- | --- | --- | --- |
|  |  |  |  |  |  |  | **MR- rCBV** | **FET-PET-rCBV** | **FET PET (20-40’)** | **MR versus PET rCBV** |
| 1 | M | 62 | L F | ME II | No | Yes | 7.98 | 9.74 | 2.62 | widely agreeing |
| 2 | M | 69 | R P | ME I | No | Yes | 10.7 | 20.5 | 2.38 | widely agreeing |
| 3 | F | 51 | R T | ME I | No | Yes | 5.36 | 9.06 | 1.81 | widely agreeing |
| 4 | M | 43 | L T | ME I | No | Yes | 3.83 | 5.65 | 2.26 | widely agreeing |

**Legends Supplemental Table 2**

*sex: F = female, M = male*

*age in years at the time of the PET and MRI study*

*L = left hemispheric, R = right hemispheric, F = frontal, P= parietal, T = temporal*

*^*^ histological diagnosis: ME I = meningioma WHO grade I, ME II = meningioma WHO grade II*

*CE in MRI = contrast enhancement in MRI*

*TBR_mean_ = mean tumour to brain ratio*

*Visual scoring: Mean value of 3 independent raters. Widely agreeing (Score 1-2), different Score (3-4)*
